# Supplementary material for: Work-related smartphone use during off-job hours and work-life conflict: A scoping review
Source: PLOS Digit Health. 2024 Jul 30;3(7):e0000554. doi: 10.1371/journal.pdig.0000554 (PMC11288435; doi:10.1371/journal.pdig.0000554)
Supplement: S2 Table — (DOCX) [file pdig.0000554.s002.docx]

**S2 Table.** Operationalisation and measurement of smartphone technology and work-life conflict in included studies, and additional study variables.

| **Study** |  | **Smartphone technology** | **Work-life conflict** | **Other study variables** |
| --- | --- | --- | --- | --- |
| Brown and Palvia [37] | Definition | **Mobile-device:** A personal device that combines a cell phone with a hand-held computer, typically offering internet access, data storage, e-mail capability, etc. (such as a smart phone) [37]. | Not defined. | Work-related mobile-device usage at work, Personal mobile-device usage at work, Productivity, Employer expectations, and Flexibility |
|  | Measurement | Mobile device-use was measured using a six-item Likert-scale. It was not clear whether the scale was self-constructed or pre-existing.  The average variance extracted (AVE) value of the scale was .68 indicating satisfactory convergent validity, and composite reliability was .93. Factor loadings for all items were greater than .6.  It was unclear whether the study assessed functional or perceptual aspects of work-related smartphone use during nonworking hours. | Measured using a five-item Likert-scale. It was not clear whether the scale was self-constructed or pre-existing.  The AVE value was .82 indicating satisfactory convergent validity, and composite reliability was .96. Factor loadings for all items were greater than .8. |  |
| Derks et al. [45] | Conceptual | **Smartphone:** a mobile device with the functionality of a pocket PC, which facilitates calendar management, unlimited access to the Internet, making phone calls, and receiving emails anytime, anywhere [45]. | **Work-home interference (WHI):** a process of negative interaction between work and home domains [1]. | Recovery activities from work: Psychological detachment, Relaxation, Mastery, and Control/ Autonomy |
|  | Measurement | Smartphone usage for work-related purposes during non-working hours was assessed using a self-reported background questionnaire. However, the exact question asked was not stated.  It was unclear whether the study assessed the functional or perceptual aspects of work-related smartphone use during non-working hours | Daily WHI was measured using the eight-item subscale of the SWING (Survey Work– home Interaction NijmeGen; [2]. Items adjusted for day-level measurement by Van Hooff et al. [1] were used. Items were rated on a five-point Likert scale. The scale demonstrated high internal consistency (α = .97). |  |
| Derks et al. [46] | Conceptual definition | **Daily work-related smartphone use during off-job time:** not defined. | **Work-family conflict:** The extent to which professional and family responsibilities are incompatible with each-other (3). | Segmentation preference, workload, daily role family performance |
|  | Measurement | Measured using a four-item scale developed by Derks and Bakker [4] (ɑ = .78).  The scale included both structural (example item: “*Today, I used my smartphone intensively during after work hours for work-related purposes*”) and perceptual (example item: “*Today, I felt obliged to respond to work-related messages during the evening hours*”) aspects of work-related smartphone use during nonworking time. | Daily work-family conflict was measured using a five-item subscale of the Work–Family Conflict Scale [5] (ɑ = .92). Items were adjusted for day-level measurement by the researchers. |  |
| Ragsdale and Hoover [38] | Definition | **Cell phones:** Portable and flexible devices for staying connected to work [38].  The researchers focused specifically on work-related cell phone (WRCP) use during nonworking hours (38) (Cell phones as access to job demands Section, p. 55). | **Work-family conflict:** an inter-role conflict in which work, and family demands, are incompatible with each-other [3]. | Cell phone attachment, work engagement, and emotional exhaustion |
|  | Measurement | Measured using a seven-item Likert scale developed by the researchers (ɑ = .95), which captured expectations related to WRCP use (e.g., “*My supervisor relied on me carrying my cell phone to contact me*”), actual use (e.g., “*I find myself using my cell phone for work at home*”), and thinking about use (e.g., “*My cell phone has become a constant reminder of work*.”) during nonworking hours.  A review of the dimensions and sample items suggested that the scale assessed both the functional as well, as the perceptual aspects of WRCP | Assessed using a nine-item Likert scale developed by Carlson et al. [6] (ɑ = .89). |  |
| Derks et al. [47] | Definition | **Daily smartphone use after work hours** was not defined by the authors. | **Work-home interference**: an inter-role conflict in which work, and family demands are incompatible with each-other [3]. | Supervisor expectations, norms set by colleagues, workload, daily work engagement |
|  | Measurement | It was measured using a four-item Likert scale developed by Derks and Bakker [4] (ɑ = .77). Items were rated on a five-point Likert scale.  The scale measured both functional and perceptual aspects of work-related smartphone use during nonworking time. | Daily WHI was measured using the eight-item subscale of the SWING (Survey Work– home Interaction NijmeGen) [2].  Items adjusted for day-level measurement by Van Hooff et al. [1] were used. Items were rated on a five-point Likert scale. The scale demonstrated high internal consistency (α = .91). |  |
| Derks and Bakker [23] | Definition | **Smartphone:** a wireless device with functions to manage the calendar, make phone calls, browse the web, and to send and receive e-mails [4]. | **Work-home interference:** an inter-role conflict in which work, and family demands are incompatible with each-other [3]. | Daily psychological detachment, daily exhaustion, daily cynicism, and daily relaxation |
|  | Measurement | Intensive smartphone use was assessed using a four-item self-constructed scale. All items were rated on a five-point Likert scale. | Daily WHI was measured using the eight-item subscale of the SWING (Survey Work– home Interaction NijmeGen) [2].  Items adjusted for day-level measurement by Van Hooff et al. [1] were used. Items were rated on a five-point Likert scale. The scale demonstrated high internal consistency (α = .88). |  |
| Carlson et al. [39] | Definition | **Mobile device:** a smartphone or an internet-enabled tablet. | **Work-to-family conflict (WFC):** a form of inter-role conflict in which the role pressures from the work and family domains are mutually incompatible in some respect [3]. | Relationship tension, spouse family-to-work conflict, spouse job satisfaction, and spouse job performance |
|  | Measurement | Mobile device use for work (by job incumbents) during family time was assessed using a three-item scale developed by Ferguson et al. [40].  All items were rated on a five-point Likert scale and the scale demonstrated satisfactory levels of internal consistency (α = .95).  The items of the scale assessed the functional dimension of mobile device use for work during family time (example item: “*How frequently do you use a mobile device to perform your job during family time?*”) | Job incumbents’ WFC levels were measured using a nine-item scale developed by Carlson et al. [6]. The scale demonstrated satisfactory internal consistency (α = .93). |  |
| Yun et al. [48] | Definition | **Office-home smartphone (OHS):** a smartphone device used for personal uses, as well as for nonpersonal, nonfamily purposes.” [48]. The researchers focused on OHS for work purposes for the purposes of this study. | **Work-to-life conflict due to OHS**: not defined. | Job stress, user resistance to OHS, segmentation culture, and segmentation preference |
|  | Measurement | Four attributes of OHS (work overload, flexibility, autonomy, and productivity) were measured by combining items from different scales (all rated on five-point Likert scale) and by translating them into Korean (if required). All the items were reported in Table 3 of the study.  Work overload was measured using a four-item scale (α = .91) [7,8]. Example item: “*After using smartphones for work purposes, I feel that the number of requests, problems, or complaints I deal with is more than what is expected*.”  Flexibility was measured using a four-item scale (α = .88) [9]. Example item: *“Since you used smartphones for work purposes, how much flexibility have you had in selecting the location where you work?”*  Autonomy was measured using a three-item scale [7,10]. Example item: “*After using smartphones for work purposes, I control the content of my job*.”  Productivity was measured using a four-item scale (α = .79) [11]. Example item: “The work use of a smartphone helps to improve the quality of my work.”  A review of items suggested that the study measured the perceptual facet of OHS. Note: Items of flexibility and autonomy subscales loaded onto the same factor in exploratory factor analysis thus, these two subscales were combined. | It was measured using a five-item scale (α = .89) [7]. All items were rated on a five-point Likert scale. An example item includes, “The work use of smartphones interferes with my home and personal life.” |  |
| Ferguson et al. [40] | Definition | **mWork:** Defined as the frequency of using a smartphone (or a tablet) with access to the internet to access and complete work tasks during leisure time [40]. | **Work-family conflict:** a form of inter-role conflict in which the role pressures from the work and family domains are mutually incompatible in some respect [3]. | Administered to incumbent (burnout, organisational commitment, and turnover intentions)  Administered to spouse (resentment towards the organisation of the job incumbent, commitment to the job incumbent’s organisation, and engagement in mWork [control variable]) |
|  | Measurement | Job incumbents’ engagement in mWork was measured using a three-item (five-point Likert) scale [12]. The scale demonstrated satisfactory levels of internal consistency (α = .94). Example item: “*How frequently do you use a mobile device to perform your job during family time*?”  A review of the items indicated that the scale measured the functional aspect of mWork. | Job incumbents’ levels of WFC were assessed by their spouses using a nine-item scale developed by Carlson et al. [6].  The scale included items related to time-based WFC (α = .92), strain-based WFC (α = .94), and behaviour-based WFC (α = .90). |  |
| Gadeyne et al. [49] | Definition | **Work-related smartphone use outside work hours:** not defined. | **Work-to-home conflict**: Resulting from competing temporal demands of different life domains and strain or stress caused by spillovers from one life domain to the other [3]. | Integration preference, integration norms, work demands, work-related PC/laptop use outside work hours, home demands, and overtime |
|  | Measurement | It was measured using a two-item (five-point Likert) scale (α = .90) [49], where participants were asked to rate how often they used their smartphones for work-related goals outside work hours. In addition, how many minutes per day they spent on average on their smartphones for work-related purposes outside work hours.  Items on the scale measured the functional aspect of work-related smartphone use outside work hours. | It was measured on a six-item scale developed by Carlson et al. [6]. Items related to time-based (α = .86) and strain-based (α = .87) work-to-home conflict were included. All items were rated on a seven-point Likert scale. |  |
| Schieman and Young [33] | Definition | **Work contact:** Defined as the frequency with which workers send and receive work-related communications (e.g. emails, phone calls, text messages) outside of regular working hours [13,14].  This study was included because phone calls and text messages can only be made/sent using a mobile phone. | **Work-to-family:** the degree to which individuals perceive that work interferes with the responsibilities and expectations of family and competes for individuals’ finite amounts of time and energy [15]. | Psychological distress, sleep issues, job autonomy, schedule control, challenging work, and job pressure. |
|  | Measurement | Work contact was measured using three items rated on a five-point Likert scale (ɑ = .78). Example item: ‘‘How often were you called about work-related matters when you were not at work?’’  A review of items indicated that the study assessed functional aspect of work-related smartphone use during nonworking hours. | WFC was measured using a four-item scale (α = .90). All items were rated on a five-point Likert scale. |  |
| Harris [41] | Definition | **Smartphone:** a device that combines a cell phone with a hand-held computer, typically offering Internet access, data storage, e-mail capability, etc. [41].  **Work life to personal life (WLPL) smartphone intrusion:** smartphone for work-related purposes during personal time. | **Work-life balance:** the degree to which an individual is equally engaged in and satisfied with their work and family role [16]. | Organisational attitudes towards smartphones use, job stress, life satisfaction, job satisfaction, personal life to work life smartphone intrusion, and personal life to work life balance |
|  | Measurement | WLPL smartphone intrusion was measured using a 14-item (five-point Likert) self-constructed scale (α = .78). Example item: *“I feel using my smartphone for work invades my personal life.”*  A review of items indicated that the scale assessed the perceptual facet of work-related smartphone use during nonworking hours.  In addition, participants were asked, *“On average how many hours a week do you spend using your smartphone for work during personal time?”* (time spent using smartphones) and *“During an average week how often do you use your smartphone for work related activities during personal time?”* (frequency of smartphone use).  These items assessed the functional aspect of work-related smartphone use during nonworking hours. | WLPL balance was measured using a 15-item (six-point Likert) scale developed by Fisher [17]. The scale demonstrated satisfactory levels of internal consistency (α = .90). |  |
| Burney [42] | Definition | **Smartphones:** compact devices that can be used for calling, messaging, mapping, and obtaining or exchanging information [18,19]. | **Work-life balance:** the extent to which, an employee feels content with their personal and professional lives [20,21]. | N/A |
|  | Measurement | Participants were asked in the demographics questionnaire if they used a personal smartphone for work purposes, a company-issued smartphone, or both.  It was not clear whether it assessed the functional or perceptual aspect of work-related smartphone use during non-working hours. | Work-life balance was measured using a four-item scale (ɑ = .84 - .89) [22]. All items were rated on a five-point Likert scale. |  |
| Ward and Steptoe-Warren [51] | Definition | BB use for work purposes during non-work hours was not operationalized by Ward and Steptoe-Warren [51]. | Work-family conflict (WFC) was operationalized as an inter-role conflict between work and family roles [23]. | Psychological well-being, perceived job control, and psychological detachment |
|  | Measurement | The frequency of BB use for work during nonwork hours was measured using the frequency subscale of the Work Connectivity Behaviour After-Hours (WCBA; α = .88) measure [24]. All items were rated on a five-point Likert scale.  The duration of BB use for work during nonwork hours was measured using the duration subscale of WCBA measure (α = .73).  The frequency as well as the duration subscales of WCBA measure assessed the functional aspect of work-related smartphone use during nonworking hours | WFC was measured using an eight-item (five-point Likert) scale developed by Kopelman et al. (ɑ = .92) [23]. |  |
| Wei and Teng [52] | Definition | **Smartphone:** mobile phone that allows one to manage their calendar, access the internet and social media, and play games [25]. | **Work-life conflict:** an inter-role conflict stemming from incompatible role pressures from work and family domains [3]. | Employment sector and work engagement |
|  | Measurement | Work-related smartphone use outside of official working hours was assessed using a four-item, using a five-point Likert, scale [4] (α .717). Example item: “*I use my smartphone intensively*.”  A review of the items suggested that the scale assessed the perceptual aspect of work-related smartphone use during non-working hours. | Work-life conflict was measured by adapting seven items (related to work interference with personal life) of a fifteen-item scale developed by Hayman [26] (ɑ = .90) |  |
| Bowen and Zhang [54] | Definition | **Work contact:** the frequency with which employees send and receive work-related communications (e.g., emails, phone calls, text messages) outside of regular working hours [27]. This study was included because phone calls and text messages can only be made/sent using a mobile phone. | Work-family conflict (WFC) was operationalized as an inter-role conflict in which work, and family demands are incompatible with each-other [3]. | Workload pressure, job autonomy, schedule control, childcare demands, household tasks, partner’s work hours, partner support, family contact, family-to-work conflict, psychological distress, alcohol use, and sleep problems |
|  | Measurement | Work contact was measured using three items rated on a five-point Likert scale (ɑ = .78) [27]. Example item: ‘‘*How often were you called about work-related matters when you were not at work*?’  A review of the items indicated that the study assessed the functional aspect of work-related smartphone use during nonworking hours. | WFC was measured using a four-item scale developed by Bowen et al. (ɑ = .91) [28]. All items were rated on a five-point Likert scale. |  |
| van Zoonen et al. [57] | Definition | Smartphones: mobile, portable, and personalized access to communication, work and social networks, and information and application resources [57]. | Work-life conflict: the negative effects of role pressures across the borders of work into life and vice versa [3,29]. | Discussing work demands with family, discussing work demands with supervisor, organisational identification, organizational tenure, and employer expectations to use communication technology outside of formal work hours |
|  | Measurement | Smartphone use after hours was measured using a self-constructed scale. The scale included the prompt, “*Think about your smart phone use outside formal work hours. How often do you use the smart phone in the following ways* [for voice conversations, sending/receiving text messages, and sending/receiving email] *to perform your work outside of formal work hours (before or after work, on weekends, during vacations)?*” The item was rated on a seven-point Likert scale, and it was related to the functional aspect of work-related smartphone use during nonworking hours. | Work-life conflict was measured using a four-item scale developed by Hayman [26] (ɑ = .91). |  |
| Bowen et al. [55] | Definition | **Work contact**: not defined. | **Work-family conflict:** not defined. | Job autonomy and control, job pressure, psychological distress, and sleep problems. |
|  | Measurement | It was measured using three items rated on a five-point Likert scale [27] (ɑ_1_ = .84; ɑ_2_ = .83). Example item: ‘‘*How often were you called about work-related matters when you were not at work*?’’  A review of items indicated that the study assessed the functional aspect of work-related smartphone use during nonworking hours. | Work-family conflict (WFC) was measured using a four-item scale developed by Bowen et al. [30] (ɑ_1_ = .91; ɑ_2_ = .90). All items were rated on a five-point Likert scale. |  |
| Bowen et al. [56] | Definition | **Work contact:** the degree to which workers send and receive work-related communications (e.g., emails, phone calls, text messages) outside of regular working hours [27].  This study was included because phone calls and text messages can only be made/sent using a mobile phone. | **Work-family conflict (WFC):** an inter-role conflict in which work, and family demands are incompatible with each-other [3]. | job autonomy and control, job pressure, psychological distress, and sleep problems. |
|  | Measurement | Work contact was measured using three items rated on a five-point Likert scale [27] (ɑ = .84). Example item: ‘‘*How often were you called about work-related matters when you were not at work?*’’  A review of items indicated that the study assessed the functional aspect of work-related smartphone use during nonworking hours. | WFC was measured using a four-item scale developed by Bowen et al. [30] (ɑ = .91). All items were rated on a five-point Likert scale. |  |
| Fender [43] | Definition | **Work extending communication (WEC):** the degree to which organizational employees are in contact via electronic communication technology with the organization, its suppliers, or clients outside of normal working hours for work-related matters. WEC includes contacts initiated by either the organization or the employee. It is typically a phone call, email, text message or an instant message via a cell or smart phone. [31].  **After-hours electronic communication (AEC)** **expectations:** the extent to which employees with electronic communication devices (i.e., cell and smart phones) believe that they are expected to be available and responsive to organizational demands after-hours via these devices [43].  **Receptive electronic communication (REC) behaviour:** the extent to which individuals engage in work-related responsive communication and associated preparatory behaviours outside of normal working hours with members of their organizations, its customers and suppliers [43].  **Electronic tethering (ET):** the extent to which an employee perceives that they are connected to the organization outside of normal working hours from an electronic communication perspective [43]. | **Work-to-family conflict (WFC):** the degree to which an employee’s work domain hinder effective participation in their family domain [32]. | Job performance, job insecurity, family-supportive organisational perceptions, predictability, and periodicity of WEC, affective attitude towards ET, and communication technology self-efficacy |
|  | Measurement | The intensity and duration of WEC was measured using a five-item self-constructed scale (ɑ = .85). Example item: “*How many work-related communications did you receive after hours in an average week*?”. A review of items in appendix B-1 (p. 242) in Fender [31] suggested that all the five items assessed the functional aspects of work-related smartphone use during nonworking hours.    AEC expectations were measured using an 8-item self-constructed scale (ɑ = .87), using a five-point scale. Example item: “*My organization expects me to answer after-hours contacts immediately*.” A review of items in appendix B-2 (p. 242) suggested that the items assessed the perceptual aspect of work-related smartphone use during non-working hours.    The frequency of engaging in REC behaviours was measured using a 14-item self-constructed scale (ɑ = .87), using a five-point Likert scale. Example item: “*Provide status reports after hours via your cell/smart phone?”* A review of items in appendix B-3 (p. 243) indicated that the scale assessed the functional aspect of work-related use of smartphone during nonworking hours.  ET was measured using a six-item self-constructed scale (ɑ = .916) using a five-point Likert scale. Example item: “My cell/smart phone is a constant tie to my work.” A review of ET items in appendix B-4 (p. 244) suggested that the scale assessed the perceptual aspect of work-related smartphone use during nonworking hours. | Time-based WFC (ɑ = .897) was measured using a 3-item scale developed by Carlson et al. [6]. |  |
| Mansour et al. [50]** | Definition | **Use of smartphone and/or tablet for work-related purposes during nonworking hours:** the intensive use of these devices to conduct business outside official working hours. | **Work-family conflict (WFC):** a form of conflict between roles in which the general demands, the time spent, and the tension created by work interfere with family responsibilities” [5]. | Work intensification |
|  | Measurement | It was measured using a five-item, using a five-point Likert scale developed by Derks & Bakker [4]; composite reliability [CR] = .92).  Example item: “*I use my smartphone and/or tablet intensively outside business hours for business reasons*.”  A review of items indicated that the scale measured the perceptual aspect of work-related smartphone use during non-working hours. | The intensification of WFC was measured using a self-constructed scale (CR = .93) comprising of items from validated scales, which were adapted to this study. All items were rated on a five-point Likert scale. Example item: “*Since the past five years, the demands of my job make it more difficult to take on my family responsibilities*.” |  |
| Alwis and Hernvall [53] | Definition | The **perceived intensity of using information and communication technologies (ICTs) at work:** not defined. | **Work-life conflict (WLC):** the degree of perceived incompatibility between work and other life roles [35]. | Segmentation preference and perceived intensity of ICTs at work |
|  | Measurement | Perceived intensity of ICTs at work was measured using an adapted version of the six-item electronic tethering (ET), seven-point Likert, scale developed by Fender [43] (ɑ = .84; CR = .88). Example item: “*I feel as though I am always available to the organisation via technological devices (my cell/smartphone/laptop)*”  This scale measured the perceptual aspect of work-related smartphone use during non-working hours. | It was measured using the modified version of Netemeyer et al. [5] by Kreiner [36] (ɑ = .92; CR = .93). All five items were rated on a seven-point Likert scale. |  |
| Moore [44] | Definition | **Smartphones:** minicomputers, allowing users to utilize email, text, and social media from the palm of their hands [44].  **After hours communications for work-related purposes** using cell phones and/or computers: not defined. | Multiple definitions of work-life balance (WLB) were stated but, no one definition was adopted as the operational definition. | Facebook Use and Job Satisfaction |
|  | Measurement | After hours communication was measured using the five-item Technology Assisted Supplemental Work (TASW) Survey [38] (ɑ = .86). All the items were rated on a five-point Likert scale. A sample item includes, “When I fall being in my work during the day, I work hard at home at night or on weekends to get caught up by using my cell phone.” The scale measured the perceptual aspect of work-related smartphone use during non-working hours. | WLB was measured using a six-item subscale of the Work-Life Balance Survey [26] called the Work Interference with Personal Life (WIPL) subscale (ɑ = .72). All items were rated on a seven-point Likert scale. |  |

*There is inconsistency in Fender [43] regarding the number of items in the AEC expectations scale – whilst the appendix (B-2, p. 242) listed 8 items, the methods chapter (p. 138) mentioned 7 items. The coefficient alpha value reported in the table are based on the 7-item scale (p. 138). **Mansour et al. [50] did not mention but they added the term, “and/or tablet” to the scale developed by Derks and Bakker [4]. Also, the scale constructed by Derks and Bakker [4] included 4 items. It is not clear if there is an error in Mansour et al. [44] or if the researchers added an item.

**References to support S3 Table**

1. Van Hooff MLM, Geurts SAE, Kompier MAJ, Taris TW. Work–home interference: How does it manifest itself from day to day? Work Stress. 2006 Apr;20(2):145–62.

2. Geurts SAE, Taris TW, Kompier MAJ, Dikkers JSE, Van Hooff MLM, Kinnunen UM. Work-home interaction from a work psychological perspective: Development and validation of a new questionnaire, the SWING. Work Stress. 2005 Oct;19(4):319–39.

3. Greenhaus JH, Beutell NJ. Sources of Conflict between Work and Family Roles. The Academy of Management Review. 1985 Jan;10(1):76–88.

4. Derks D, Bakker AB. Smartphone Use, Work-Home Interference, and Burnout: A Diary Study on the Role of Recovery. Applied Psychology. 2014 Jul;63(3):411–40.

5. Netemeyer RG, Boles JS, McMurrian R. Development and validation of work–family conflict and family–work conflict scales. Journal of Applied Psychology. 1996;81(4):400–10.

6. Carlson DS, Kacmar KM, Williams LJ. Construction and Initial Validation of a Multidimensional Measure of Work–Family Conflict. J Vocat Behav [Internet]. 2000;56(2):249–76. Available from: https://www.sciencedirect.com/science/article/pii/S000187919991713X

7. Ahuja, Chudoba, Kacmar, McKnight, George. IT Road Warriors: Balancing Work-Family Conflict, Job Autonomy, and Work Overload to Mitigate Turnover Intentions. MIS Quarterly. 2007;31(1):1–17.

8. Moore JE. One Road to Turnover: An Examination of Work Exhaustion in Technology Professionals. MIS Quarterly [Internet]. 2000;24(1):141–68. Available from: http://www.jstor.org/stable/3250982

9. Hilbrecht M, Shaw SM, Johnson LC, Andrey J. ‘I’m Home for the Kids’: Contradictory Implications for Work–Life Balance of Teleworking Mothers. Gend Work Organ [Internet]. 2008;15(5):454–76. Available from: https://onlinelibrary.wiley.com/doi/abs/10.1111/j.1468-0432.2008.00413.x

10. Beehr TA. Perceived situational moderators of the relationship between subjective role ambiguity and role strain. Journal of Applied Psychology. 1976 Feb;61(1):35–40.

11. Tarafdar M, Tu Q, Ragu-Nathan BS, Ragu-Nathan TS. The Impact of Technostress on Role Stress and Productivity. Journal of Management Information Systems [Internet]. 2007;24(1):301–28. Available from: http://www.jstor.org/stable/40398890

12. Diaz I, Chiaburu DS, Zimmerman RD, Boswell WR. Communication technology: Pros and cons of constant connection to work. J Vocat Behav [Internet]. 2012;80(2):500–8. Available from: https://www.sciencedirect.com/science/article/pii/S000187911100114X

13. Schieman S, Glavin P. Trouble at the border?: Gender, flexibility at work, and the work-home interface. Soc Probl. 2008;55(4):590–611.

14. Voydanoff P. Work, Family, and Community: Exploring Interconnections. Routledge; 2007.

15. Greenhaus JH, Parasuraman S. A Work-Nonwork Interactive Perspective of Stress and Its Consequences. J Organ Behav Manage. 1987 Jul 7;8(2):37–60.

16. Greenhaus JH, Collins KM, Shaw JD. The relation between work–family balance and quality of life. J Vocat Behav. 2003 Dec;63(3):510–31.

17. Fisher GG. Work /personal life balance: A construct development study. [Ann Arbor]: Bowling Green State University; 2001.

18. Barkhuus L, Polichar VE. Empowerment through seamfulness: smart phones in everyday life. Pers Ubiquitous Comput. 2011 Aug 2;15(6):629–39.

19. Burg-Brown S. Work-Life Balance. Journal of Property Management [Internet]. 2013 [cited 2024 Feb 20];49–53. Available from: https://journalpm.s3.us-east-2.amazonaws.com/wp-content/uploads/2021/09/07193030/July-August-2013.pdf

20. Evans L, Young G. Work–life balance and welfare. Australasian Psychiatry. 2017 Apr 10;25(2):168–71.

21. Malik M, Wan D, Dar L, Akbar A, Naseem MA. The Role Of Work Life Balance In Job Satisfaction And Job Benefit. Journal of Applied Business Research (JABR). 2014 Oct 21;30(6):1627–38.

22. Brough P, Timms C, O’Driscoll MP, Kalliath T, Siu OL, Sit C, et al. Work–life balance: a longitudinal evaluation of a new measure across Australia and New Zealand workers. The International Journal of Human Resource Management. 2014 Oct 28;25(19):2724–44.

23. Kopelman RE, Greenhaus JH, Connolly TF. A model of work, family, and interrole conflict: A construct validation study. Organ Behav Hum Perform [Internet]. 1983;32(2):198–215. Available from: https://www.sciencedirect.com/science/article/pii/0030507383901472

24. Richardson KM, Thompson CA. High Tech Tethers and Work-family Conflict: A Conservation of Resources Approach. Engineering Management Research. 2012 Apr 27;1(1):29–43.

25. Derks D, Bakker AB, Peters P, van Wingerden P. Work-related smartphone use, work–family conflict and family role performance: The role of segmentation preference. Human Relations. 2016 May 14;69(5):1045–68.

26. Hayman J. Psychometric Assessment of an Instrument Designed to Measure Work Life Balance. Research and Practice in Human Resource Management [Internet]. 2005 [cited 2024 Feb 20];13(1):85–91. Available from: https://citeseerx.ist.psu.edu/document?repid=rep1&type=pdf&doi=97154bd8cd2b901d6bbd29ca9f7f196c5e0fbd68

27. Schieman S, Young MC. Are communications about work outside regular working hours associated with work-to-family conflict, psychological distress and sleep problems? Work Stress [Internet]. 2013;27(3):244–61. Available from: https://doi.org/10.1080/02678373.2013.817090

28. Bowen P, Govender R, Edwards P. Validation of the Schieman and Young measurement scales for work contact, work-family conflict, working conditions, psychological distress and sleep problems in construction industry professionals. BMC Public Health [Internet]. 2018;18(1):1199. Available from: https://doi.org/10.1186/s12889-018-6100-7

29. Kahn RL, Quinn RP. Role stress: A framework for analysis. In: McLean A, editor. Occupational mental health. New York: Rand McNally; 1970. p. 50–115.

30. Bowen P, Govender R, Edwards P, Cattell K. Work-related contact, work–family conflict, psychological distress and sleep problems experienced by construction professionals: an integrated explanatory model. Construction Management and Economics. 2018 Mar 4;36(3):153–74.

31. Fender CM. Electronic Tethering: Perpetual Wireless Connectivity to the Organization. Drexel University; 2010.

32. Voydanoff P. Work Demands and Work-to-Family and Family-to-Work Conflict. J Fam Issues. 2005 Sep 30;26(6):707–26.

33. Keeney J, Boyd EM, Sinha R, Westring AF, Ryan AM. From “work–family” to “work–life”: Broadening our conceptualization and measurement. J Vocat Behav [Internet]. 2013;82(3):221–37. Available from: https://www.sciencedirect.com/science/article/pii/S0001879113000274

34. Kreiner GE. Consequences of work-home segmentation or integration: a person-environment fit perspective. J Organ Behav [Internet]. 2006;27(4):485–507. Available from: https://onlinelibrary.wiley.com/doi/abs/10.1002/job.386

35. Fenner GH, Renn RW. Technology-assisted supplemental work and work-to-family conflict: The role of instrumentality beliefs, organizational expectations and time management. Human Relations [Internet]. 2010;63(1):63–82. Available from: https://doi.org/10.1177/0018726709351064
