# Supplementary material for: Work-related smartphone use during off-job hours and work-life conflict: A scoping review
Source: PLOS Digit Health. 2024 Jul 30;3(7):e0000554. doi: 10.1371/journal.pdig.0000554 (PMC11288435; doi:10.1371/journal.pdig.0000554)
Supplement: S3 Table — (DOCX) [file pdig.0000554.s003.docx]

**S3 Table.** Summary of findings: The association between the use of smartphone technology for work in off-job hours and work-life conflict.

| Study | Relationship Found (Yes/No) | Summary of Main findings (reported effect size) | Data Analysis |
| --- | --- | --- | --- |
| [37] | Yes | Work-related mobile device usage at home was significantly related to work-life conflict (β = .301, p < .01). | Partial Least Squares Structural Equation Modelling (PLS-SEM) |
| [45] | No | The difference between levels of work-home interference reported by smartphone users and PC users was not statistically significant, F(1, 78) < 1. | Multilevel modelling |
| [46] | Yes | Daily work-related smartphone use during off-job time was negatively related to daily work-family conflict, γ = -.48, p < .001. | Multilevel modelling |
| [38] | Yes | Work-related cell phone use during nonworking hours was positively associated with work-family conflict, β = .42, p < .01. | Hierarchical regression |
| [47] | Yes | Daily smartphone use after work hours was positively related to daily work-home interference, γ = .291, p < .001. | Multilevel modelling |
| [23] | Yes | Daily smartphone use was positively related to daily work-home interference in two statistical models: (i) γ = .227, p < .01, and (ii) γ = .334, p < .001. | Multilevel modelling |
| [39] | Yes | Job incumbent mobile device use for work during family time was positively related to job incumbent work-family conflict, β = .20, p < .01. | Structural equation modelling |
| [48] | Mixed | Work-to-life conflict was significantly predicted by only one aspect of office home smartphone – work overload, β = .67, p < .001. It was not significantly related to flexibility (effect size not reported) and productivity (effect size not reported). | Covariance based structural equation modelling |
| [40] | Yes | Engagement in work by the job incumbent was positively associated with time-based work-family conflict (β = .38, p<.05), strain-based work-family conflict (β = .35, p < .05), and behaviour-based work-family conflict (β = .17, p < .05). | Structural Equation Modelling |
| [49] | No | Work-related smartphone use outside work hours was not related to time-based work-to-home conflict (β = -.051, p > .05) and strain-based work-to-home conflict (β = .002, p > .05). Work-related PC/laptop use after work hours was related to time-based work-to-home conflict (β = .169, p < .01). It was, however, not related to strain-based work-to-home conflict (β = .101, p > .05). | Hierarchical regression analysis |
| [33] | Yes | Work contact was positively associated with work-to-family conflict, β = .310, p < .001. | Hierarchical regression analysis |
| [41] | Yes | Work life to personal life (WLPL) smartphone intrusion was negatively related to WLPL balance, r = -.598, p < .01. Time spent using a smartphone for work during personal time was negatively related to WLPL balance, r = -.339, p < .01. Frequency of smartphone use for work during personal time was negatively related to WLPL balance, r = -.261, p < .01. | Bivariate correlation |
| [42] | Yes | There was a statistically significant difference in work-life balance of participants who used a personal smartphone, a company-issued smartphone, or both for work, F (2, 159) = 11.67, p < .001. Results of post-hoc analysis showed that managers who used a personal smartphone (mean difference = 2.298, p < .05) or a company-issued smartphone (mean difference = 3.651, p < .05) reported better work-life balance than those who used both. | One-way ANOVA and Tukey’s HSD test |
| [51] | Yes | The frequency of BlackBerry use for work purposes during nonwork hours was positively related to work-family conflict, r = .34, p < .01. The duration of BlackBerry use for work purposes during nonwork hours was positively related to work-family conflict, r = .30, p < .01. | Bivariate correlation and mediation analysis |
| [52] | Yes | Work-related smartphone use outside official working hours was positively related to work-life conflict, β = .40, p < .001. | Linear regression |
| [54] | Yes | Work contact was positively associated with work-family conflict, β = .21, p < .001. | Structural equation modelling |
| [57] | No | Smartphone use after formal work hours was not related to work-life conflict, B = -.004, p = .986. | Structural equation modelling |
| [55] | Yes | Work contact was positively related to work-family conflict (n_1_ = 311, r = .46, p < .001). | Bivariate correlation |
| [56] | Yes | Work contact was positively related to work-family conflict: β = .223, p < .001. | Structural equation modelling |
| [43] | Yes | Work extending communication ( β = .172, p < .05), after-hours electronic communication expectations (β = .320, p < .001), and receptive electronic communication behaviours (β = .115, p < .05) were positively related to time-based WFC. The relationship between electronic tethering and time-based work-family conflict was not examined. | Structural equation modelling |
| [50] | Yes | Work-related use of smartphone and/or tablet outside working hours was positively related to work-family conflict (B = .14, p < .01). | Structural equation modelling |
| [53] | Yes | The perceived intensity of using information and communication technologies (cell phone, smartphone, or laptop) was positively related to work-life conflict (β = .51, p < .01). | Structural equation modelling |
| [44] | Yes | Technology assisted supplemental work using cell phone or computer was positively associated with work interference with personal life (r = .20 p < .05). | Bivariate correlation |
