# Supplementary material for: Work-related smartphone use during off-job hours and work-life conflict: A scoping review
Source: PLOS Digit Health. 2024 Jul 30;3(7):e0000554. doi: 10.1371/journal.pdig.0000554 (PMC11288435; doi:10.1371/journal.pdig.0000554)
Supplement: S4 Table — (DOCX) [file pdig.0000554.s004.docx]

**S4 Table.** Variables moderating or mediating the relationship between smartphone-use and work-life conflict.

| Study | Moderator/Mediator | Findings |
| --- | --- | --- |
| [46] | Moderator | Segmentation preference positively moderated the negative relationship between daily work-related smartphone use during off-job time and daily work-family conflict, γ = .54, p < .001. Results of simple slope tests showed that for participants with high segmentation preference (‘segmenters’), the relationship between work-related smartphone use during off-job time and work-family conflict was positive but, not statistically significant, β = .25, p > .05. In contrast, for participants with low segmentation preference (integrators), the relationship between the two variables was negative and significant: β = -1.21, p < .001. |
| [38] | Moderator | Work-related cell phone use during nonworking hours was positively associated with work-family conflict: β = .42, p < .01. Cell phone attachment was negatively associated with work-family conflict (β = -.16, p < .01) and negatively moderated the positive relation between work-related cell phone use and work-family conflict: β = -.93, p < .01. |
| [47] | Moderator | Daily smartphone use after work hours was positively related to daily work-home interference (WHI): γ = .272, p < .001. Supervisor expectations were positively related to daily work-home interference (γ = .128, p < .05) and it positively moderated the positive association between daily smartphone use after work hours and daily work-home interference: γ = .102, p < .05. |
| [47] | Moderator | Daily smartphone use after work hours was positively related to daily work-home interference: γ = .251, p < .001. Norms set by colleagues were positively related to daily work-home interference: γ = .231, p < .01. The positive association between daily smartphone use after work hours and daily work-home interference was not moderated by norms set by colleagues: γ = .072, p > .05. |
| [47] | Moderator | Daily smartphone use after work hours was positively related to daily work-home interference: γ = .290, p < .001. Daily work engagement was negatively related to daily work-home interference (γ = -.270, p < .001) and it negatively moderated the positive relationship between daily smartphone use after work hours and daily work-home interference: γ = -.198, p < .001. |
| [49] | Moderator | Integration preference was negatively related to time-based work-to-home conflict (β = -.208, p < .01) and strain-based work-to-home conflict (β = -.211, p < .01). However, it did not moderate the relationship between work-related smartphone use after work hours and time-based work-to-home conflict (β = .024, p > .05) and between work-related smartphone use after work hours and strain-based work-to-home conflict (β = .005, p > .05). |
| [49] | Moderator | Integration norms were positively related to time-based work-to-home conflict (β = .110, p < .05) and strain-based work-to-home conflict (β = .156, p < .01). However, it did not moderate the relationship between work-related smartphone use after work hours and time-based work-to-home conflict (β = -.090, p > .05) and between work-related smartphone use after work hours and strain-based work-to-home conflict (β = -.098, p > .05). |
| [49] | Moderator | The three-way interaction among work-related smartphone use after work hours, integration preference, and integration norms was not related to time-based work-to-home conflict (β = -.087, p > .05) and strain-based work-to-home conflict (β = -.082, p > .05). |
| [49] | Moderator | Work demands were positively associated with time-based work-to-home conflict (β = .230, p < .01) and strain-based work-to-home conflict (β = .231, p < .01). However, it did not moderate the relationship between work-related smartphone use after work hours and time-based work-to-home conflict (β = -.044, p > .05) and between work-related smartphone use after work hours and strain-based work-to-home conflict (β = -.022, p > .05). |
| [49] | Moderator | The three-way interaction among work-related smartphone use after work hours, integration preference, and work demands was not related to time-based work-to-home conflict (β = .007, p > .05) and strain-based work-to-home conflict (β = -.037, p > .05). |
| [33] | Moderator | Job autonomy was negatively related to work-to-family conflict (β = -.099, p < .001) and it negatively moderated the positive relationship between work contact and work-to-family: β = -.060, p < .001. |
| [33] | Moderator | Some schedule control was negatively related to work-to-family (β = -.134, p < .001), but it did not moderate the positive relationship between work contact and work-to-family: β = -.058, p > .05. |
| [33] | Moderator | Full schedule control was negatively related to work-to-family (β = -.219, p < .001) and it negatively moderated the positive relationship between work contact and work-to-family: β = -.124, p < .01. |
| [33] | Moderator | Challenging work was negatively related to work-to-family (β = -.137, p < .001) and it negatively moderated the positive relationship between work contact and work-to-family: β = -.065, p < .01. |
| [33] | Moderator | Job pressure was positively related to work-to-family (β = .406, p < .001) and it positively moderated the positive relationship between work contact and work-to-family: β = .031, p < .01. |
| [41] | Moderator | Work life to personal life smartphone intrusion was not related to work life to personal life balance, β = .04, p = .898. Organisation’s attitude towards smartphone use was not related to work life to personal life balance: β = .23, p = .370. However, organisation’s attitude towards smartphone uses negatively moderated the impact of work life to personal life smartphone intrusion on work life to personal life balance: β = -.231, p = .014. |
| [51] | Mediators | Job control and psychological detachment from work mediated the relationship between frequency of BlackBerry use for work purposes during non-working hours and work-family conflict (WFC), indirect effect = .1435, 95% BC bootstrap CIs (.0522, .2472). Job control and psychological detachment from work mediated the relationship between duration of BB use for work purposes during nonwork hours and work-family conflict, indirect effect = .3347, 95% BC bootstrap CIs (.0236, .6838).  Job control did not mediate the relationship between frequency of BlackBerry use for work purposes during nonwork hours and WFC, indirect effect = .0080, 95% BC bootstrap CIs (-.0071, .0436). It also did not mediate the relationship between duration of BlackBerry use for work purposes during nonwork hours and WFC, indirect effect = .0081, 95% BC bootstrap CIs (-.0648, .1173). In contrast, psychological detachment mediated the relationship between frequency of BlackBerry use for work purposes during nonwork hours and work-family conflict, indirect effect = .1355, 95% BC bootstrap CIs (.0521, .2355). Also, it mediated the relationship between duration of BlackBerry use for work purposes during nonwork hours and work-family conflict, indirect effect = .3266, 95% BC bootstrap CIs (.0598, .6441).  A comparison of indirect effects of both the mediators revealed that the indirect effects of psychological detachment from work were larger than the indirect effects of job control for both the frequency of BlackBerry use for work during nonwork hours (-.3185, 95% BC bootstrap CIs [-.6150, -.0690]) and duration of BlackBerry use for work during nonwork hours (-.1276, 95% BC bootstrap CIs [-.2244, -.0454]). |
| [52] | Moderator | The study examined employment sector (public vs. private) as a moderator in the relationship between work-related smartphone use outside official working hours and work-life conflict but, there were significant omissions in the results section (e.g., significance values for β values were not reported), which made it impossible to determine whether the analysis was significant or not. Although the researchers claimed that the moderation effect was not significant, the presented data is incomplete to confirm this assertion. |
| [57] | Mediator | The total effect of smartphone use after work hours on work-life conflict was not significant: B = -.010, p = .889. The direct effect of smartphone use after work hours on work-life conflict was not significant, B = -.004, p = .986. The direct effect of smartphone use after work hours on communication about family demands with one’s supervisor was significant, B = .139, p = .026. The direct effect of communication about family demands with one’s supervisor on work-life conflict was significant: B = -.113, p = .048. The indirect effect for the mediating role of communication about family demands with one’s supervisor in the relationship between smartphone use after work hours and work-life conflict was significant: B = -.016, p = .027. |
| [57] | Mediator | The direct effect of smartphone use after work hours on communication about work demands with family members was not significant, B = .127, p = .076. The direct effect of communication about work demands with family members on work-life conflict was not significant: B = .084, p = .137. The indirect effect for the mediating role of communication about work demands with one’s family members in the relationship between smartphone use after work hours and work-life conflict was not significant: B = .011, p = .111. |
| [43] | Mediator | Receptive electronic communication behaviour partially mediated the relationship between work extending communication and time-based work-family conflict. The direct effect of receptive electronic communication behaviour on time-based work-family conflict (B = .135, p = .042) was less than the total effect of receptive electronic communication behaviour on time-based work-family conflict (B = .217, p = .001). |
| [43] | Mediator* | Receptive electronic communication behaviour fully mediated the relationship between work extending communication and strain-based work-family conflict. The direct effect of work extending communication on strain-based work-family conflict (B = .037, p =.588) was not less than the total effect (B = .126, p = .050) and not statistically significant. |
| [43] | Mediator** | Receptive electronic communication behaviour did not mediate the relationship between after-hours communication expectations and time-based work-family conflict. The direct effect of after-hours communication expectations on time-based work-family conflict (B = .338, p < .001) was less than the total effect of after-hours communication expectations on time-based work-family conflict (B = .392, p < .001). |
| [43] | Mediator** | Receptive electronic communication behaviour did not mediate the relationship between after-hours communication expectations and strain-based work-family conflict. The direct effect of after-hours communication expectations on strain-based work-family conflict (B = .272, p < .001) was less than the total effect of after-hours communication expectations on time-based work-family conflict (B = .330, p < .001). |

*It is not clear why the author [43] claimed full mediation in table 17 (p. 274) especially when the total effect in the examined model was not statistically significant – the p-value for the relationship between REC behaviour and strain-based WFC was not less than 0.05. **Based on the values reported in table 17 (p. 274), there is clear evidence for partial mediation effect of REC behaviour in the relation between AEC expectations and time-based WFC, and for the partial mediation effect of REC behaviour in the relation between AEC expectations and strain based WFC. The direct effect (c’ path) was less than the total effect (c path) in both the models thus, partial mediation can be claimed but it is not clear why the authors have mentioned “No mediation” for both the paths.
