# Supplementary material for: Work-related smartphone use during off-job hours and work-life conflict: A scoping review
Source: PLOS Digit Health. 2024 Jul 30;3(7):e0000554. doi: 10.1371/journal.pdig.0000554 (PMC11288435; doi:10.1371/journal.pdig.0000554)
Supplement: S5 Table — (DOCX) [file pdig.0000554.s005.docx]

**S5 Table.** Summary of findings examining work-related wellbeing, attitudes, and work behaviours as outcomes.

| Study | Relationship Found | Examined outcome(s) | Findings |
| --- | --- | --- | --- |
| **Positive Wellbeing** | | | |
| [45] | Mixed | Psychological detachment | Work-home interference was not significantly associated with psychological detachment, γ = .012, p > .05. Smartphone-use was not significantly associated with psychological detachment, γ = -.113, p > .05. However, smartphone-use negatively moderated the positive relationship between work-home interference and psychological detachment, γ = -.134, p < .001. |
| [45] | Yes | Relaxation | Work-home interference was positively related to relaxation, γ = .118, p < .05. Smartphone-use was negatively related to relaxation, γ = -.48, p < .001. Smartphone-use negatively moderated the positive relation between work-home interference and relaxation, γ = -.226, p < .001. |
| [45] | Mixed | Mastery | Work-home interference was not significantly associated with mastery, γ = .086, p >.05. Smartphone-use was negatively associated with mastery, γ = -.248, p < .05. Smartphone-use negatively moderated the positive relationship between work-home interference and mastery, γ = -.128, p < .001. |
| [45] | Yes | Control/Autonomy | Work-home interference was positively associated with control/autonomy, γ = .200, p < .01. Smartphone-use was negatively associated with control/autonomy, γ = -.378, p < .01. Smartphone-use negatively work-home interference the positive relationship between WHI and control/autonomy, γ = -.229, p < .001. |
| [38] | Yes | Work engagement | Work-related cell phone use during nonworking hours was positively related to work engagement, β = .21, p < .01. Cell phone attachment was positively related to work engagement (β = .40, p < .01) and positively moderated the positive relationship between work-related cell phone use and work engagement (β = 1.57, p < .01). |
| [39] | Yes | Spouse job satisfaction | The indirect effect for the mediating roles of job incumbent WFC, relationship tension, and spouse FWC in the relationship between job incumbent MD use for work during family time and spouse job satisfaction was negative and significant, indirect effect = -.008, 95% CIs (-.022, -.003). The indirect effect for the same path excluding the mediating role of relationship tension was also significant, indirect effect = -.008, 95% CIs (-.021, -.001). |
| [41] | Mixed | Life satisfaction | WLPL balance (r = .171, p < .05) and frequency of smartphone use for work during personal time (r = .139, p < .05) were related to life satisfaction. WLPL smartphone intrusion (r = -.029, p > .05) and time spent on smartphone for work during personal time (r = .029, p > .05) were not related to life satisfaction. |
| [41] | Mixed | Job satisfaction | WLPL smartphone intrusion (r = -.223, p < .01), WLPL balance (r = .311, p < .01), and frequency of smartphone use for work during personal time (r = .161, p < .05) were related to job satisfaction. Time spent on smartphone for work during personal time (r = -.033, p > .05) was not related to job satisfaction. |
| [41] | Yes | Personal life to work life (PLWL) balance | WLPL smartphone intrusion (r = -.551, p < .01), WLPL balance (r = .753, p < .01), time spent on smartphone for work during personal time (r = -.416, p < .01), and frequency of smartphone use for work during personal time (r = -.315, p < .01) were related to PLWL balance. |
| [51] | Mixed | Well-being | Neither frequency (r= -.03; p > .05) nor duration (r= .01; p > .05) of BlackBerry (BB) use for work during nonwork hours was significantly related to well-being.  Job control and psychological detachment from work mediated the relationship between frequency of BB use for work during nonwork hours and wellbeing, indirect effect = -.0615, 95% BC bootstrap CIs (-.1323, -.0081). However, it did not mediate the relationship between duration of BB use for work during nonwork hours and wellbeing, indirect effect = -.1307, 95% BC bootstrap CIs (-.3666, .0456). A comparison of indirect effects of both the mediators revealed no significant difference in degrees for either the frequency of BB use for work during nonwork hours (.0349, 95% BC bootstrap CIs [-.0275, .1053]) or the duration of BB use for work during nonwork hours (.0959, 95% BC bootstrap CIs [-.0503, .2796]).  Job control did not mediate the relationship between frequency of BB use for work during nonwork hours and wellbeing (indirect effect = -.0133, 95% BC bootstrap CIs [-.0647, .0076]) and between duration of BB use for work during nonwork hours and wellbeing (indirect effect = -.0174, 95% BC bootstrap CIs [-.1693, .0739]). In contrast, psychological detachment mediated the relationship between both frequency of BB use for work during nonwork hours and wellbeing (-.0482, 95% BC bootstrap [-.1172, -.0047]) as well as between duration of BB use for work and wellbeing (-.1133, 95% BC bootstrap [-.3035, -.0086]). |
| [52] | Yes | Work engagement | Results of simple linear regression revealed that work-related smartphone use outside official working hours was positively related to work engagement, β = .28, p < .001.  The study examined employment sector (public vs. private) as a moderator in the relationship between work-related smartphone use outside official working hours and work engagement but, there were significant omissions in the results section (e.g., significance values for β values were not reported), which made it impossible to determine whether the analysis was significant or not. Although the researchers claimed that the moderation effect was not significant, the presented data is incomplete to confirm this assertion. |
| [43] | Yes | Job satisfaction | Work-to-home segmentation preference (β = -.296, p < .001) and psychological and physiological strain (β = -.343, p < .001) negatively associated with job satisfaction. |
| [44] | Mixed | Job satisfaction | Technology assisted supplemental work using cell phone or computer was positively associated with job satisfaction (r = .16, p < .05). The correlation between Facebook use and job satisfaction was not statistically significant (r = -.0001, ns). |
| **Negative Wellbeing** | | | |
| [38] | Yes | Emotional exhaustion | Work-related cell phone use during nonworking hours was not significantly associated with emotional exhaustion, β = -.02, p > .05. Cell phone attachment was negatively related to emotional exhaustion (β = -.37, p < .01) and negatively moderated the negative relationship between work-related cell phone use and emotional exhaustion, β = -1.38, p < .01. |
| [23] | Mixed | Exhaustion | Daily exhaustion was significantly predicted by daily work-home interference (γ = .350, p < .001). It was not significantly predicted by daily smartphone use (γ = .037, p > .05). However, daily smartphone use positively moderated the positive relationship between daily work-home interference and daily exhaustion, γ = .083, p < .05. |
| [23] | Mixed | Cynicism | Daily cynicism was significantly predicted by daily work-home interference (γ = .214, p < .001). It was not significantly predicted by daily smartphone use (γ = -.036, p > .05). The moderating effect of daily smartphone use in the relationship between daily work-home interference and daily cynicism was not significant, γ = .035, p > .05. |
| [39] | Yes | Relationship tension | Job incumbent work-to-family conflict was positively related to relationship tensions between job incumbents and spouses, β = .52, p < .01. The indirect effect for the mediating role of job incumbent work-to-family conflict in the relationship between job incumbent mobile-device (MD) use for work during family time and relationship tension between partners was significant, indirect effect = .102, 95% CIs (.044, .186). |
| [39] | Yes | Spouse family-to-work conflict | Job incumbent work-to-family conflict was positively related to spouse FWC, β = .14, p < .10. Relationship tension between partners was positively related to spouse FWC, β = .29, p < .001. The indirect effect for the mediating roles of job incumbent WFC and relationship tension in the relationship between job incumbent MD use for work during family time and spouse FWC was significant, indirect effect = .029, 95% CIs (.013, .061). |
| [48] | Mixed | Job stress | Work-to-life conflict significantly predicted job stress, β = .54, p < .001. The work overload aspect of OHS was not significantly related to job stress (effect size not reported). |
| [40] | Mixed | Job incumbent burnout | Job incumbent burnout was positively predicted by strain-based work-family conflict (WFC), β = .47, p < .01. Time-based WFC (β = -.04, p > .01) and behaviour-based WFC (β = .04, p > .01) were not significantly associated with job incumbent burnout. |
| [33] | Yes | Psychological distress | Work contact was positively related to psychological distress, β = .117, p < .001. Job autonomy (β = -.083, p < .001), some schedule control (β = -.085, p < .001), full schedule control (β = -.109, p < .01), challenging work (β = -.145, p < .001), and job pressure (β = .197, p < .001) were associated with psychological distress. Full schedule control negatively moderated the positive relationship between work contact and psychological distress, β = -.082, p < .01. Work-to-family (WFC) was positively associated with psychological distress, β = .236, p < .001. |
| [33] | Yes | Sleep problems | Work contact was positively associated with sleep problems, β = .123, p < .001. Job autonomy (β = -.096, p < .001), some schedule control (β = -.104, p < .01), challenging work (β = -.169, p < .001), and job pressure (β = .180, p < .001) were associated with sleep problems. Job autonomy negatively moderated the positive relationship between work contact and sleep problems, β = -.062, p < .01. Challenging work negatively moderated the positive relationship between work contact and sleep problems, β = -.067, p < .01. Job pressure positively moderated the positive relationship between work contact and sleep problems, β = .035, p < .01. WFC was positively associated with sleep problems, β = .255, p < .001. |
| [41] | Mixed | Job stress | Work life to personal life (WLPL) smartphone intrusion (r = .204, p < .01), WLPL balance (r = -.542, p < .01), time spent on using a smartphone for work during personal time (r = .205, p < .01) were related to job stress. Frequency of smartphone use for work during personal time (r = .061, p > .05) was not related to job stress. |
| [41] | Yes | Personal life to work life (PLWL) smartphone intrusion | WLPL smartphone intrusion (r = .588, p <.01), WLPL balance (r = -.497, p < .01), time spent on smartphone for work during personal time (r = .295, p < .01), and frequency of smartphone use for work during personal time (r = .213, p < .01) were related to PLWL smartphone intrusion. |
| [54] | Mixed | Family-to-work conflict (FWC) | Work-to-family conflict (WFC; β = .26, p < .001), family contact (β = .20, p < .001), household tasks (β = .21, p < .001), childcare demands (β = .20, p < .001), and partner support (β = -.28, p < .001) were related to FWC. Partner’s work hours (β = .01 [not significant]) was not related to FWC. |
| [54] | Mixed | Alcohol use | FWC (β = -.12, p < .01) and sleep problems (β = .23, p < .01) were related to alcohol use. WFC was not related to alcohol use, β = -.08 (not significant). |
| [54] | Mixed | Sleep problems | WFC (β = .13, p < .05) and psychological distress (β = .58, p < .001) were positively associated with sleep problems. FWC was not related to sleep problems (β = .00 [not significant]). |
| [54] | Yes | Psychological distress | WFC (β = .49, p < .001) and FWC (β = .27, p < .001) were positively related to psychological distress. |
| [56] | Yes | Psychological distress | Psychological distress was related to job pressure (β = .286, p < .001), work-family conflict (β = .320, p < .001), and work experience (β = -.158, p < .001). |
| [56] | Yes | Sleep problems | In the first path model, work-family conflict (β = .282, p < .001), job pressure (β = .155, p < .001), and work contact (β = .088, p = .030) were positively related to sleep problems. In the second path model, work-family conflict (β = .111, p = .004), work contact (β = .071, p = .036), and psychological distress (β = .546, p < .001) were related to sleep problems. |
| [43] | Yes | Psychological and physiological strain | ET (β = .157, p < .01), time-based WFC (β = .317, p < .001), and work-to-home segmentation preference (β = .215, p < .001) were positively associated with psychological and physiological strain. |
| **Attitudes** | | | |
| [37] | Yes | Employer expectations | Work-related mobile-device usage at home was significantly related to employer expectations, β = .349, p < 0.01. |
| [48] | Yes | User resistance to office home smartphone (OHS) | Work-to-life conflict significantly predicted user resistance to OHS, β = .23, p < .001. The work overload aspect of OHS significantly predicted user resistance to OHS, β = .26, p < .01. |
| [40] | Mixed | Job incumbent organizational commitment | Job incumbent burnout was negatively related to job incumbent organizational commitment, β = -.20, p < .01. Engagement in mWork by job incumbent was not significantly related to their organizational commitment, β = .01, p > .05. Spousal resentment towards job incumbent’s organization was not related to job incumbent organizational commitment, β = .01, p > .05. Spousal commitment to job incumbent’s organisation was positively related to job incumbent organizational commitment, β = .54, p < .05. |
| [40] | Yes | Spousal resentment towards job incumbent’s organization | Job incumbent’s time-based WFC (β = .29, p < .05), strain-based WFC (β = .29, p < .05), and behaviour-based WFC (β = .15, p < .05) were positively related to spousal resentment towards job incumbent’s organization. Spousal engagement in mWork was also significantly associated with spousal resentment towards job incumbent’s organization, β = -.08, p < .05. |
| [40] | Yes | Spousal commitment to job incumbent’s organization | Job incumbent burnout (β = -.27, p < .05) and spousal resentment towards job incumbent’s organization (β = -.14, p <.05) were negatively related to spousal commitment to job incumbent’s organization. Spousal engagement in mWork was also significantly associated with spousal commitment to job incumbent’s organization, β = .08, p < .01. |
| [40] | Mixed | Job incumbent’s turnover intentions | Job incumbent organizational commitment (β = -.28, p < .01), job incumbent organizational tenure (β = -.03, p < .01), and spousal commitment to job incumbent’s organization (β = -.64, p < .05) were negatively related to job incumbent’s turnover intentions.  The following path models from job incumbent mWork to job incumbent’s turnover intentions were significant: (i) job incumbent mWork predicted job incumbent’s turnover intentions via job incumbent strain-based WFC, job incumbent burnout, and job incumbent organizational commitment, indirect effect = .009, 95% CIs (.003, .026); (ii) job incumbent mWork significantly predicted job incumbent’s turnover intentions via job incumbent time-based WFC, spousal resentment towards job incumbent’s organization, and spousal commitment to job incumbent’s organization, indirect effect = .010, 95% CIs (.003, .027); (iii) job incumbent mWork significantly predicted job incumbent’s turnover intentions via job incumbent strain-based WFC, spousal resentment towards job incumbent’s organization, and spousal commitment to job incumbent’s organization, indirect effect = .009, 95% CIs (.003, .024); (iv) job incumbent mWork significantly predicted job incumbent’s turnover intentions via job incumbent behaviour-based WFC, spousal resentment towards job incumbent’s organization, and spousal commitment to job incumbent’s organization, indirect effect = .004, 95% CIs (.001, .015); (v) job incumbent mWork significantly predicted job incumbent’s turnover intentions via job incumbent’s strain-based WFC, job incumbent burnout, and spousal commitment towards job incumbent’s organization, indirect effect = .028, 95% CIs (.012, .066), and; (vi) job incumbent mWork significantly predicted job incumbent’s turnover intentions via job incumbent’s strain-based WFC, job incumbent burnout, spousal commitment to organizational commitment, and job incumbent’s organizational commitment, indirect effect = .007, 95% CIs (.002, .018). |
| [57] | Mixed | Organisational identification | Smartphone use after formal work hours (β = .137, p < .05) and communication about family demands with supervisor (β = .135, p < .05) were positively related to organizational identification. Communication about work demands with family was not related to organisational identification, β = .059 p > .05.  There was a significant indirect effect relationship between smartphone use after formal work hours and organisational identification through communication about family demands with supervisors, β = .019, p = .018. However, the indirect effect relationship between smartphone use after formal work hours and organisational identification through communication about work demands with family members was not significant, β = .008, p = .148. |
| [43] | Mixed | Affective organisational commitment | Electronic tethering (ET; β = .159, p < .001), time-based work-to-family (WFC; β = .099 p < .05), and job satisfaction (β = .652, p < .001) were positively associated with affective organisational commitment. ET instrumentality strengthened the positive relationship between ET and affective organisational commitment (β = .088, p < .05). The relationship between ET instrumentality and time-based WFC was not statistically significant (β = .012, ns). |
| **Behaviour** | | | |
| [37] | No | Productivity | Work-related mobile-device usage at home was not significantly related to productivity, β = .061, p > 0.05. |
| [46] | Mixed | Family role performance | Daily work-related smartphone use during off-job time was positively related to relationship dimension of daily family-role performance (e.g., providing emotional support to family members), γ = .39, p < .05. Information about the relationship between daily work-related smartphone use during off-job time and task dimension of daily family role performance was not provided.  The positive relationship between daily work-related smartphone use and daily family role performance was negatively moderated by segmentation preference, γ = -.28, p < .05.  Daily work-family conflict was negatively related to the relationship dimension of daily family role performance, γ = -.31, p < .001.  Results of mediated moderation analysis showed that in comparison with participants with high segmentation preference, those with low segmentation preference reported better daily family-role performance (relationship dimension) on days they more intensively used their smartphones for work-related purposes during off-job time, via reduced daily work-family conflict. |
| [39] | Yes | Spouse job performance | The indirect effect for the mediating roles of job incumbent WFC, relationship tension, and spouse FWC in the relationship between job incumbent MD use for work during family time and spouse job performance was negative and significant, indirect effect = -.006, 95% CIs (-.015, -.002). The indirect effect for the same path excluding the mediating role of relationship tension was also significant, indirect effect = -.005, 95% CIs (-.016, -.001). |
| [40] | Yes | Engagement in mWork by job incumbent | Job incumbent organizational commitment was positively related to engagement in mWork, β = .36, p < .05. |
| [57] | Yes | Communication about family demands with supervisor | Smartphone use after formal work hours was positively related to communication about family demands with supervisor, β = .139, p < .05. |
| [57] | Yes | Communication about work demands with family | Smartphone use after formal work hours was positively related to communication about work demands with family, B = .139, p = .026. |
| [43] | Yes | Job performance | Receptive electronic communication (REC) behaviour (β = .278, p < .001), time-based WFC (β = .164, p < .01), and job satisfaction (β = .154, p < .05) were positively associated with job performance. Psychological and physiological strain was negatively associated with job performance (β = -.147, p < .001). The relationship between ET and job performance was not significant (β value not reported). |
