# Supplementary material for: Work-related smartphone use during off-job hours and work-life conflict: A scoping review
Source: PLOS Digit Health. 2024 Jul 30;3(7):e0000554. doi: 10.1371/journal.pdig.0000554 (PMC11288435; doi:10.1371/journal.pdig.0000554)
Supplement: S1 Text — (DOCX) [file pdig.0000554.s006.docx]

**S1 Text.** Example search strategy for PsycINFO

S1: smartphones OR (mobile devices) OR (mobile phone) OR (cell phone) OR iPhone OR blackberry OR (android phone) OR (windows phone)

S2: (work-life conflict) OR (work-family conflict) OR (work-life balance) OR (work-life interface) OR (work-life interference)

S1 yielded 19,012 results and S2 yielded 7,004 results. For both the searches, the identified terms should have appeared "anywhere" in a manuscript. When both S1 and S2 were combined ([S1] AND [S2]), it produced 51 outputs, and after date restrictions (01/01/2012 - 29/11/2023) were imposed, it produced 44 outputs.
