# Supplementary material for: Work-related smartphone use during off-job hours and work-life conflict: A scoping review
Source: PLOS Digit Health. 2024 Jul 30;3(7):e0000554. doi: 10.1371/journal.pdig.0000554 (PMC11288435; doi:10.1371/journal.pdig.0000554)
Supplement: Attachment — Submitted filename: Response to reviewers.docx [file pdig.0000554.s007.docx]

26 May 2024

Dear Editor,

Thank you for the opportunity to submit the following revised manuscript to be considered for publication in PLOS Digital Health:

**Work-related smartphone use during off-job hours and work-life conflict: a scoping review.**

Holly Blake, Juliet Hassard, Jasmeet Singh, Kevin Teoh.

**Editor’s comments**

**The paper titled "Work-related Smartphone Use During Off-Job Hours and Work-Life Conflict: A Scoping Review" is well-written and interesting.**

Thank you for this positive comment.

**In order to enhance the rigor and comprehensiveness of their study, it is imperative for the authors to meticulously detail their methodology, offering transparency and clarity to their research process. Additionally, the authors can explore deeper certain concepts. Moreover, it is essential for the authors to frame their research in a context that highlights its potential impact on policymaking and future investigations - by elucidating the practical implications and broader significance of their findings, the authors can underscore the relevance and urgency of their work in informing policy decisions and guiding future research.**

Thank you for these comments – we have endeavoured to provide a point-by-point response to each reviewer below which we believe has addressed these issues.

**Reviewer 1**

**This is work that was carried out using the Prisma methodology. In general, the work meets the requirements of this methodology.**

Thank you for this positive comment.

**A better systematization of Table 1 Characteristics of Included Studies is suggested**

This has been actioned. We have split the table into two, one remains as Table 1 (with significantly reduced text), the other is a new Supplementary file S3 which contains the detail on operationalisation and additional study variables. All Supplementary files have been renumbered accordingly. Since that has reduced the length of the paper, we have updated S1 Table (PRISMA checklist) with the new page numbers.

**line 246 (n = 1 (37); one-week data collection period),**

We have removed ‘one-week data collection period’.

**Reviewer 2**

**This is an interesting scoping review on the use of the smart phone in off-work hours. The topic is important and timely, and the paper is very well written.**

Thank you for this positive comment.

**Although it is described like this in the framework of Arksey and O’Malley, I assume that Step 1 would be redundant as the research question has been clear before the authors selected the method.**

Thank you for this comment. We have used the Arksey and O’Malley framework which is a six-stage methodological framework. Stage 1 is identification of the research question. Since this framework has been followed, we have reported each stage accordingly. However, we have clarified that the research question was developed following a search of the literature.

We have changed the word ‘step’ for ‘stage’ to be consistent with other published studies using the framework. Reporting the research question for stage 1 is in line with other published research using this methodology:

Chen Y, Ding J, Xi Y, Huo M, Mou Y, Song Y, Zhou H, Cui X. Thirst in heart failure: A scoping review. Nurs Open. 2023 Aug;10(8):4948-4958. doi: 10.1002/nop2.1818.

Dionisi, S.; Giannetta, N.; Di Simone, E.; Ricciardi, F.; Liquori, G.; De Leo, A.; Moretti, L.; Napoli, C.; Di Muzio, M.; Orsi, G.B. The Use of mHealth in Orthopedic Surgery: A Scoping Review. Int. J. Environ. Res. Public Health 2021, 18, 12549. https://doi.org/10.3390/ijerph182312549

**Also, aims are described in the introduction of the paper which differ from the research question in Step 1.**

Thank you for this observation. To clarify this, we have removed any redundant or repeated text.

The statement in the introduction now refers only to the aim of the study (to conduct a scoping review). The detail in Stage 1 includes only the research question and objectives (for the review).

**Could you please describe how you selected the databases? Why did you not include Pubmed for example?**

Given the research question and subject area for this study (smart-phone use during off-job hours), we selected databases primarily from the social sciences e.g., psychology, business, management. Due to limited resources it was not possible to include every possible database, and PubMed was not included here since it focuses primarily on biomedical and life sciences literature (as described here <https://pubmed.ncbi.nlm.nih.gov/about/#:~:text=PubMed%20Overview,and%20abstracts%20of%20biomedical%20literature> ). It was deemed that most relevant papers would be captured within the selected social sciences databases, although we recognise that some papers could have been missed. This has been added to the limitations.

**Why did you exclude qualitative studies?**

This was due to the focus of our research question: “What is the *association* between the use of smartphone technology for work purposes in off-job hours and employees’ self-reported work-life conflict?”. We therefore were looking for studies that (clearly operationalised and) measured the impact of work-related smartphone use during off-job hours quantifying associations using measurement tools. We documented the measurement tools used in the included studies.

Only three qualitative studies were excluded based on our searches, as per the PRISMA diagram. However, we have now referred to the exclusion of qualitative studies within the study limitations:

We intentionally excluded qualitative studies due to the nature of our research question and study aims, however, a qualitative or mixed-methods review may provide additional insights into this subject area.

**-       Did you include protocols and ongoing research projects or only completed studies?**

No – we included published, completed studies and protocols were excluded. This has been clarified in the search strategy (and limitations of the study in the discussion).

**-       Please include some more details about the stakeholders. How were they selected? What impact did the stakeholder consultations have on the results?**

Stakeholder consultation is an optional stage (Stage 6) in Arksey and O’Malley framework. Stakeholders were selected through professional networks. As per the adopted framework, the stakeholders were involved in establish research priorities and engaged in a brainstorming activity focused on knowledge translation.

More details have been provided in the ‘Stage 6’ sub-section.

We produced recommendations for employers and line managers with stakeholder input during review Stage 6. These were already outlined in ‘Review implications’ but have also now been included in a new figure: Fig.3.

**Table 1 is quite extensive. Could some of the criteria be broken down and reported in a more structured way?**

We have split the table into two, one remains as Table 1 (with significantly reduced text), the other is a new Supplementary file S3 which contains the detail on operationalisation and additional study variables. All Supplementary files have been renumbered accordingly. Since that has reduced the length of the paper, we have updated S1 Table (PRISMA checklist) with the new page numbers.

**Reviewer 3**

**The study follows a clear structure and methodological framework, and the findings are well-described.**

Thank you for this positive comment.

**Some terms, such as "psychological detachment from work," could be defined or explained briefly for readers who may not be familiar with them.**

Thank you for pointing this out – we agree that some terms may not be familiar to all readers. We have therefore added new descriptions of terms including:

Smartphones, work-home interference, smartphone-induced brain-drain, job control, job autonomy, integration preference, integration norms, and psychological detachment from work.

**A section on future research directions based on the findings of this scoping review can be added.**

Thank you for the suggestion – we have amended a sub-section heading to:

“Review implications for research and practice”

Under this sub-section we had previous presented one suggestion for future research but have now added several more.

**It's good that Google Scholar was also used for additional articles, but a brief mention of the criteria used for selecting articles from Google Scholar to maintain consistency with the articles obtained from other databases.**

Thank you for this observation. This has been included within the search strategy.

**While revising your submission, please upload your figure files to the Preflight Analysis and Conversion Engine (PACE) digital diagnostic tool.**

Figures have been checked using this diagnostic tool.
.
